# Endogenous GAS6 and Mer receptor signaling regulate prostate cancer stem cells in bone marrow

**DOI:** 10.18632/oncotarget.8365

**Published:** 2016-03-25

**Authors:** Younghun Jung, Ann M. Decker, Jingcheng Wang, Eunsohl Lee, Lulia A. Kana, Kenji Yumoto, Frank C. Cackowski, James Rhee, Peter Carmeliet, Laura Buttitta, Todd M. Morgan, Russell S. Taichman

**Affiliations:** ^1^ Department of Periodontics and Oral Medicine, University of Michigan School of Dentistry, Ann Arbor, MI, USA; ^2^ Department of Internal Medicine, Division of Hematology and Oncology, University of Michigan School of Medicine, Ann Arbor, MI, USA; ^3^ Laboratory of Angiogenesis and Vascular Metabolism, Vesalius Research Center (VRC), VIB, K.U. Leuven, Belgium; ^4^ Laboratory of Angiogenesis and Vascular Metabolism, Department of Oncology, K.U. Leuven, Belgium; ^5^ Department of Molecular, Cellular and Developmental Biology, University of Michigan, Ann Arbor, MI, USA; ^6^ Department of Urology, University of Michigan School of Medicine, Ann Arbor, MI, USA

**Keywords:** endogenous GAS6, Mer, prostate cancer, cancer stem cells, bone marrow

## Abstract

GAS6 and its receptors (Tryo 3, Axl, Mer or “TAM”) are known to play a role in regulating tumor progression in a number of settings. Previously we have demonstrated that GAS6 signaling regulates invasion, proliferation, chemotherapy-induced apoptosis of prostate cancer (PCa) cells. We have also demonstrated that GAS6 secreted from osteoblasts in the bone marrow environment plays a critical role in establishing prostate tumor cell dormancy. Here we investigated the role that endogenous GAS6 and Mer receptor signaling plays in establishing prostate cancer stem cells in the bone marrow microenvironment.

We first observed that high levels of endogenous GAS6 are expressed by disseminated tumor cells (DTCs) in the bone marrow, whereas relatively low levels of endogenous GAS6 are expressed in PCa tumors grown in a *s.c.* setting. Interestingly, elevated levels of endogenous GAS6 were identified in putative cancer stem cells (CSCs, CD133+/CD44+) compared to non-CSCs (CD133–/CD44–) isolated from PCa/osteoblast cocultures *in vitro* and in DTCs isolated from the bone marrow 24 hours after intracardiac injection. Moreover, we found that endogenous GAS6 expression is associated with Mer receptor expression in growth arrested (G_1_) PCa cells, which correlates with the increase of the CSC populations. Importantly, we found that overexpression of GAS6 activates phosphorylation of Mer receptor signaling and subsequent induction of the CSC phenotype *in vitro* and *in vivo*.

Together these data suggest that endogenous GAS6 and Mer receptor signaling contribute to the establishment of PCa CSCs in the bone marrow microenvironment, which may have important implications for targeting metastatic disease.

## INTRODUCTION

Prostate cancer (PCa) is the second leading cause of cancer deaths in American males [1, 2]. Death of most PCa patients is associated with metastasis to bone [3]. Therefore understanding the mechanisms that are involved in metastasis to bone and the survival of disseminated tumor cells (DTCs) in marrow are crucial for the development of novel therapeutic strategies.

Growth arrest-specific 6 (GAS6), a ligand for Tyro3, Axl, and Mer or TAM family of receptors, is a molecule with a broad range of immunologic and growth regulating activities in many different tissue types. Gas6 was originally isolated from quiescent fibroblasts [4] and GAS6 expression is enhanced during quiescent cell states [5]. Previously we demonstrated GAS6 signaling regulates invasion, proliferation, and chemotherapy-induced apoptosis of PCa cells [6]. We also found that GAS6 secreted from osteoblasts plays a critical role in establishing PCa cell dormancy [6]. Moreover, GAS6 signaling inhibits PCa proliferation, suggesting that once the DTCs enter the niche, interactions between GAS6 and its receptors may regulate PCa dormancy [6]. We further demonstrated that differential expression of TAM receptors is associated with a molecular switch between a dormant and a proliferative phenotype in PCa metastases [7].

Cancer stem cells (CSCs) are thought to play a critical role in the establishment and maintenance of neoplasms and the ability of metastases to gain footholds in distant organs. It remains unclear whether microenvironmental signals contribute to the maintenance of CSCs once they arrive in target tissues or whether niche signals promote the expression of a CSC phenotype by DTCs [6–10]. Although these interactions have been under intense study, how DTCs become CSCs in the bone marrow is not well understood. Several studies suggest that GAS6 or GAS6 signaling may be linked to the CSC phenotype. For example, recent work has shown that high levels of GAS6, expressed in many human tumors, correlates with decreased survival [11–13]. Moreover GAS6 mRNA is upregulated in mammospheres from primary human mammary epithelial cells grown in anchorage-independent culture conditions [14], and the loss of Gas6 expression in GAS6 knockout mice leads to a reduction in neural stem-like cell numbers within the sub-ventricular zone (SVZ) of the adult mammalian brain [15]. Expression of Mer is also known to participate in the activation of several oncogenic signaling pathways in several tumor types [16]. Mer receptor signaling is known to block IL-3-dependent proliferation, leading to G_1_/S arrest in murine 32D cells [17]. Mer expression is also crucial for the maintaining primary glioblastoma-derived tumor spheres and increases expression of Nestin and Sox2 in a glioblastoma spheroid culture model [18]. In addition, inhibition of Mer receptor signaling results in reduced colony formation and a decrease of tumor volume in a human melanoma murine xenograft model [19].

We hypothesized that endogenous GAS6 expression and Mer receptor signaling may regulate the cell cycle and induction of a CSC phenotype by PCa cells in the bone marrow microenvironment. Consistent with this, we show that high levels of endogenous GAS6 expression were found in DTCs recovered from the bone marrow and the levels were increased in the CSC population. We also show that endogenous GAS6 is associated with Mer receptor expression in growth arrested G_1_ phase during the cell cycle, which is correlated with induction of a CSC phenotype. Finally, we demonstrate that GAS6 overexpression activates phosphorylation of the Mer receptor, leading to an increase in the number of CSCs among DTCs recovered from the bone marrow. Our results suggest that the activation of Mer receptor signaling by endogenous GAS6 contributes to the establishment and/or maintenance of PCa CSCs in the bone marrow microenvironment.

## RESULTS

### Endogenous GAS6 expression is observed in PCa cells in the bone marrow microenvironment

**Figure 1:**
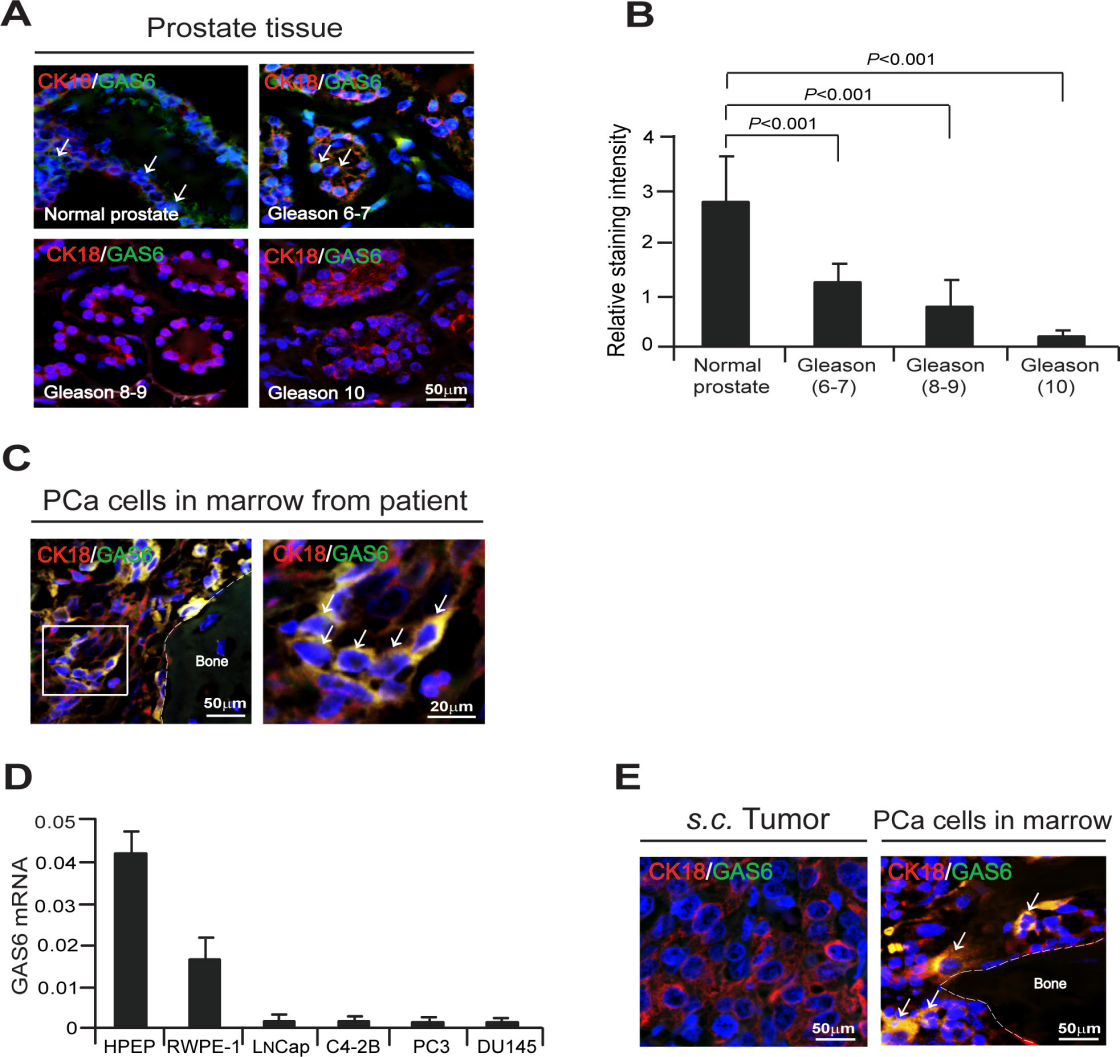
Bone marrow microenvironment activates endogenous GAS6 expression in PCa cells (**A**) GAS6 (green) expression in cytokeratin 18 (CK18, red) expressing cells (white arrows) in human prostate tissue microarrays (TMAs) as detected by immunofluorescence staining. Blue, DAPI nuclear stain. Bar = 50 μm. TMAs are normal prostate tissue (*n* = 9), Gleason 6–7 prostate cancer tissue (*n* = 10), Gleason 8 prostate cancer tissue (*n* = 8), and Gleason 9–10 prostate cancer tissue (*n* = 18). (**B**) Quantification of Figure 1A. Data represent as mean ± s.d. (Student's *t*-test). (**C**) Left panel: GAS6 (green) expression in CK18 (red) expressing PCa cells in bone marrow of a PCa patient as detected by immunofluorescence staining. Blue, DAPI nuclear stain. Bar = 50 μm. Right panel: GAS6 (green)/CK18 (red) positive cells (white arrows) in the magnification of the white rectangle from left panel. Bar = 20 μm. (**D**) mRNA levels of GAS6 expression in PCa cells as quantified by real-time PCR. Human prostate epithelial cell lines, HPEP and RWPE-1 were used as a control. (**E)** GAS6 (green) expression in CK18 (red) expressing PCa cells (white arrows) in *s.c.* PCa tumors in SCID mice and PCa cells in bone marrow from *s.c.* PCa tumors in SCID mice as detected by immunofluorescence staining. Blue, DAPI nuclear stain. Bars = 50 μm.

To explore the role that GAS6 plays in tumor progression, we examined the extent to which GAS6 expression is correlated with PCa progression in men who underwent radical prostatectomy for localized prostate cancer. Human prostate tissue microarrays (TMAs) were comprised of both cancer and adjacent normal prostate tissue from prostatectomy specimens, and tumors were categorized as low/intermediate grade (Gleason 6–7), and high grade (Gleason 8–10). TMAs were examined for the expression of GAS6 by immunofluorescence staining. High expression of GAS6 was detected in normal prostate tissues, while a decrease of GAS6 expression correlated with tumor aggressiveness (Figure 1A, 1B). Strikingly, substantial numbers of GAS6 expressing PCa cells were detected in the bone marrow of PCa patients (Figure 1C). In addition, we examined the expression levels of GAS6 in PCa cell lines. Under normal culture conditions, relatively low levels of GAS6 mRNA expression were observed in human PCa cells (Figure 1D), and GAS6 secretion was not detected in the conditioned media used to culture these cells (data not shown). Next we examined the expression of GAS6 in human PCa cells grown subcutaneously (*s.c.*) in SCID mice and in DTCs present in the bone marrow of these mice by immunofluorescence staining. We were unable to detect expression of GAS6 in PCa tumors grown in a *s.c.* setting. However, expression of GAS6 was detected in DTCs present in the bone marrow which had been shed from the *s.c.* PCa tumors (Figure 1E). Together, these findings suggest that the bone marrow microenvironment alters expression of GAS6 by PCa cells.

### PCa CSCs (CD133+/CD44+) express high levels of GAS6 in the bone marrow microenvironment

**Figure 2:**
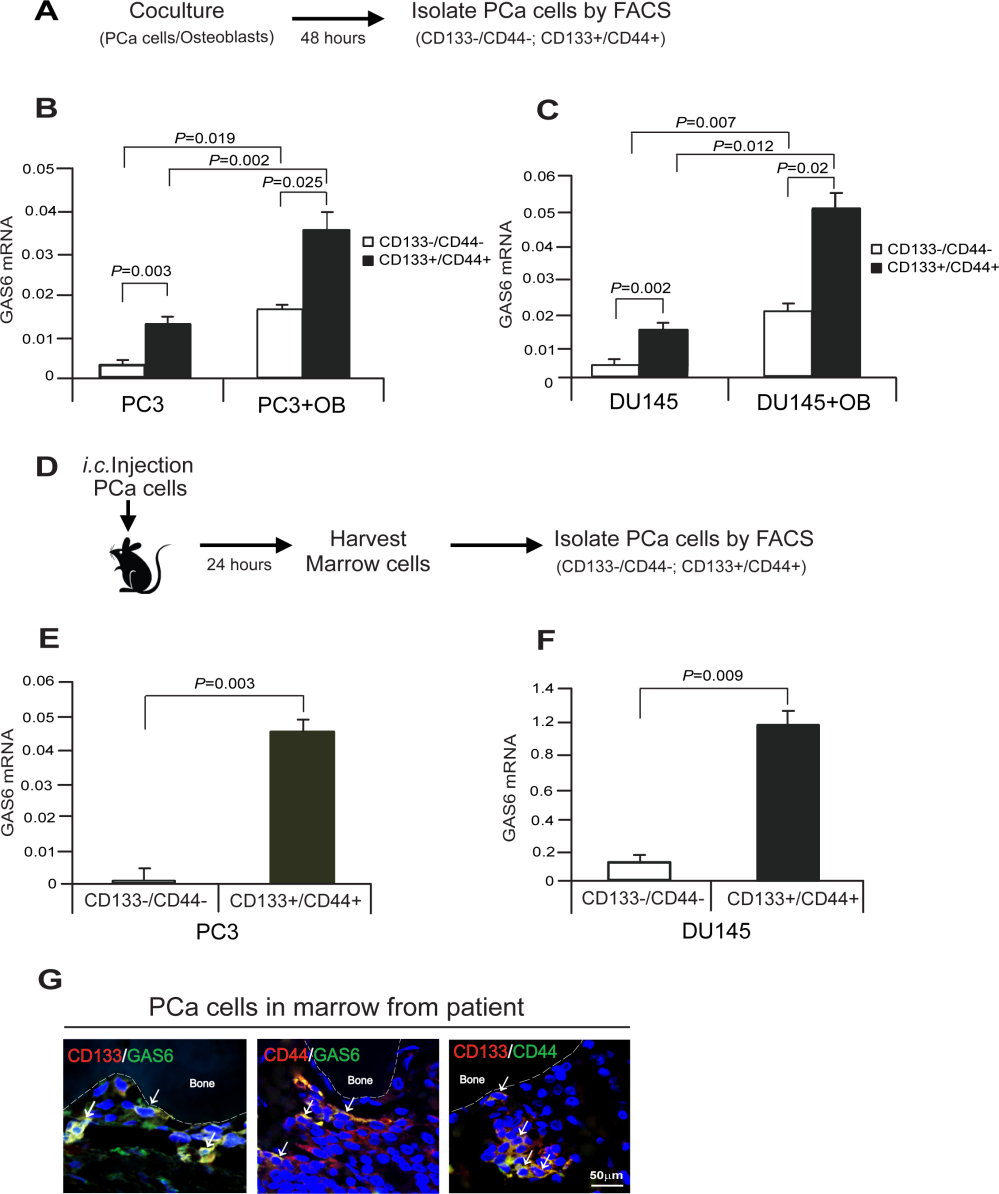
Cancer stem cells express high level of GAS6 in PCa cells in bone marrow microenvironment (**A**) Experimental model of isolation of PCa CSC cells *in vitro* cocultures of PCa cells with osteoblasts (OB). (**B, C**) Expression of GAS6 mRNA in CD133–/CD44– or CD133+/CD44+ populations from the cocultures of PCa cells with osteoblasts *in vitro* as quantified by real-time PCR. Data are representative of mean with s.d. (Student's *t*-test). (**D**) Experimental model of isolation of PCa CSC cells in marrow *in vivo*. (**E, F**) Expression of GAS6 mRNA in CD133–/CD44– or CD133+/CD44+ populations from DTCs in bone marrow at 24 hours after *i.c.* injection of PCa cells in SCID mice as quantified by real-time PCR. Data are representative of mean with s.d. (Student's *t*-test). (**G**) Immunofluorescence staining of CD133 (red)/GAS6 (green), CD44 (red)/GAS6 (green), or CD133 (red)/CD44 (green) (white arrows) in PCa cells in bone marrow of a PCa patient. Blue, DAPI nuclear stain. Bar = 50 μm.

To explore whether different phenotypic populations of PCa cells express different levels of GAS6 in the bone marrow microenvironment, PCa cells were segregated based upon expression of CD133 and CD44 from cocultures with osteoblasts *in vitro*, and then examined for GAS6 expression (Figure 2A). We found that GAS6 expression was significantly elevated in CD133+/CD44+ populations compared with CD133–/CD44– populations (Figure 2B, 2C). Moreover, GAS6 expression was significantly more pronounced in PCa cells that were cocultured with osteoblasts versus those cultured in the absence of osteoblasts (Figure 2B, 2C). Based upon the *in vitro* results, *in vivo* studies were performed to assess the same question. For these studies, *i.c.* injection of PCa cells into SCID mice was performed and 24 hours later the PCa cells present in the bone marrow were segregated based on CD133 and CD44 expression and evaluated for GAS6 mRNA expression (Figure 2D). In line with the *in vitro* results, higher levels of GAS6 expression were observed in the CD133+/CD44+ population compared with CD133–/CD44– cells recovered from the bone marrow (Figure 2E, 2F). Using immunofluorescence staining, we next examined GAS6 expression in PCa cells identified in human marrow coexpressing CD133 or CD44. Here GAS6 expression was positively correlated with both of the CD133 and CD44 markers (Figure 2G). Collectively, these data suggest that the bone marrow microenvironment plays a significant role in the regulation of GAS6 by PCa cells, and in particular by CD133 and CD44 expressing CSC populations.

### Growth arrested cells by the associating endogenous GAS6 and Mer receptor correlate with CSC populations in PCa cells

**Figure 3:**
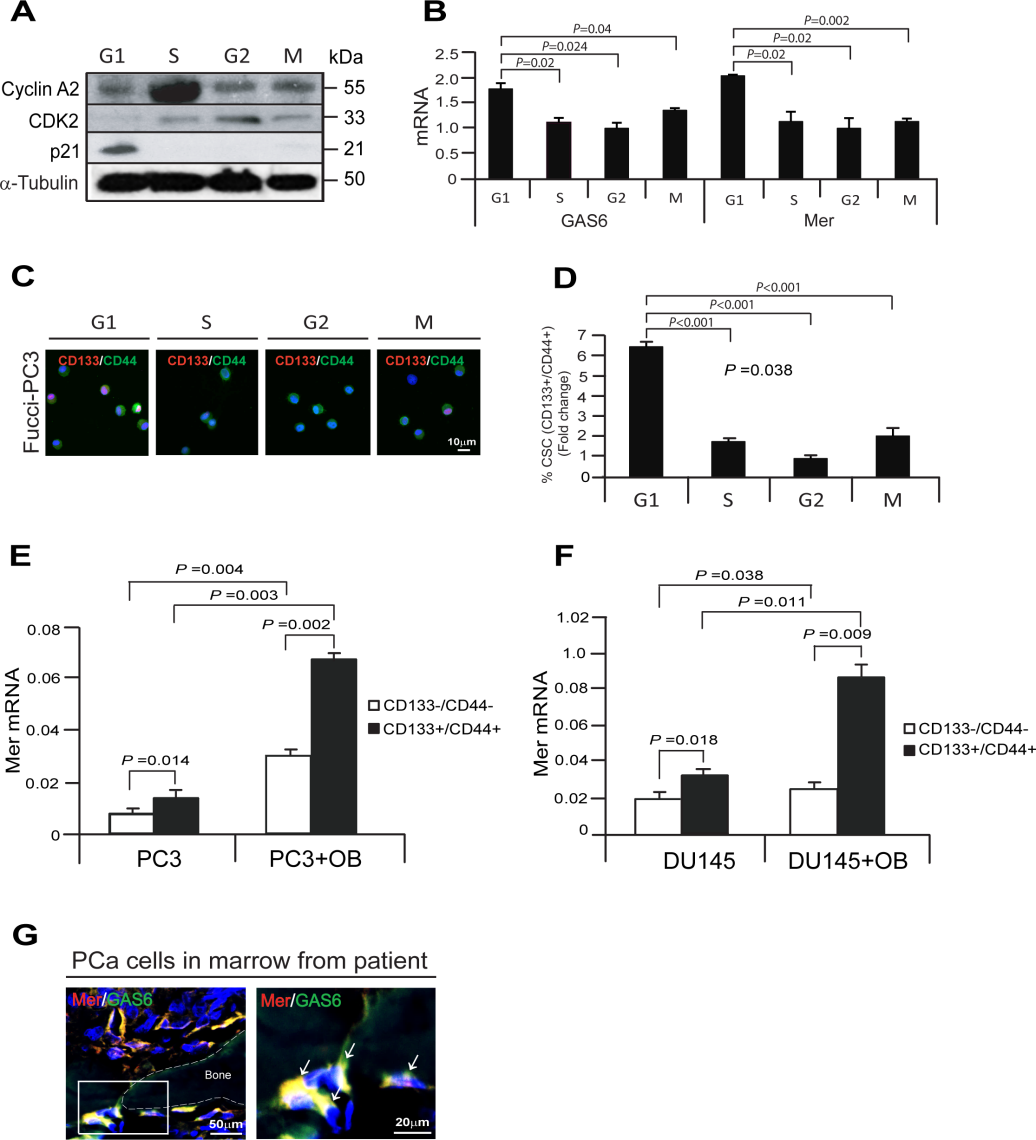
Growth arrested cells by the associating with GAS6 and Mer receptor correlate with CSC populations in PCa cells (**A**) Expression of the cell-cycle specific protein markers corresponding to G_1_, S, G_2_, or M phase in Fucci-PC3 cells as evaluated by Western blot. (**B**) GAS6 or Mer mRNA expression in G_1_, S, G_2_, or M phase in Fucci-PC3 cells as quantified by real-time PCR. Data are representative of mean with s.d. (Student's *t*-test). (**C**) Immunofluorescence staining of CD133 (red)/CD144 (green) in G_1_, S, G_2_, or M phase in Fucci-PC3 cells. Blue, DAPI nuclear stain. Bar = 10 μm. (**D**) Quantification of Figure 3C. Data are representative of mean with s.d. (Student's *t*-test). (**E, F**) Mer mRNA expression in CD133–/CD44– or CD133+/CD44+ in coculture PCa cells (PC3, DU145) with osteoblasts as quantified by real-time PCR. Data are representative of mean with s.d. (Student's t-test). (**G**) Left panel: Immunofluorescence staining of Mer (red)/GAS6 (green) in PCa cells in bone marrow of a PCa patient. Blue, DAPI nuclear stain. Bar = 50 μm. Right panel: Mer (red)/GAS6 (green) positive cells (white arrows) in the magnification of the white rectangle from left panel. Bar = 20 μm.

GAS6 and TAM receptor signaling are involved in the regulation of cell growth and survival (4, 5, 10, 15–19, 33). Previous investigations have demonstrated that G_1_/S arrest is dependent on Mer receptor signaling (17). We therefore explored the possibility that Mer receptor signaling may be responsible for the growth arrest induced by GAS6 in PCa cells. For these investigations, the cell-cycle specific Fucci-vectors were employed in PC3 cells and Fucci expression was used to isolate cells at different stages of the cell cycle [21]. Western blots were then used to validate the cell cycle status of the PCa cells isolated based upon Fucci expression (Figure 3A). We next examined GAS6 or Mer mRNA expression in the cell-cycle specific phases of Fucci-PC3 cells *in vitro*. We found that GAS6 and Mer mRNA expression are highly elevated in G_1_ phase compared with S, G_2_, or M phase of Fucci-PC3 cells, suggesting the association between GAS6 and Mer receptor expression during the cell cycle (Figure 3B). We further examined whether CSC populations are correlated with a quiescent phase of the cell cycle in PCa cells. Immunofluorescence staining demonstrates increased numbers of CD133/CD44 positive cells in G_1_ phase compared with S, G_2_, or M phase in PCa cells (Figure 3C, 3D). To explore whether the bone marrow microenvironment also supports high levels of Mer expression in CSCs, we examined Mer expression in CD133–/CD44– and CD133+/CD44+ populations from PCa cells co-cultured with osteoblasts *in vitro* (Figure 2A). We found significantly higher levels of Mer mRNA expression in CD133+/CD44+ populations compared with CD133–/CD44–. In addition, Mer mRNA expression was significantly more pronounced in CD133+/CD44+ populations isolated from cocultures of PCa cells with osteoblasts compared with CD133+/CD44+ cells cultured alone (Figure 3E, 3F). Finally, Mer expression was closely associated with GAS6 expression in PCa cells in the bone marrow from a PCa patient by immunofluorescence staining (Figure 3G). These data suggest that expression of GAS6 and Mer receptor is associated with the growth arrest of PCa cells, which also correlates with the numbers of CSC populations.

### GAS6 overexpression increases CSCs through the activation of Mer receptor signaling in PCa cells

**Figure 4:**
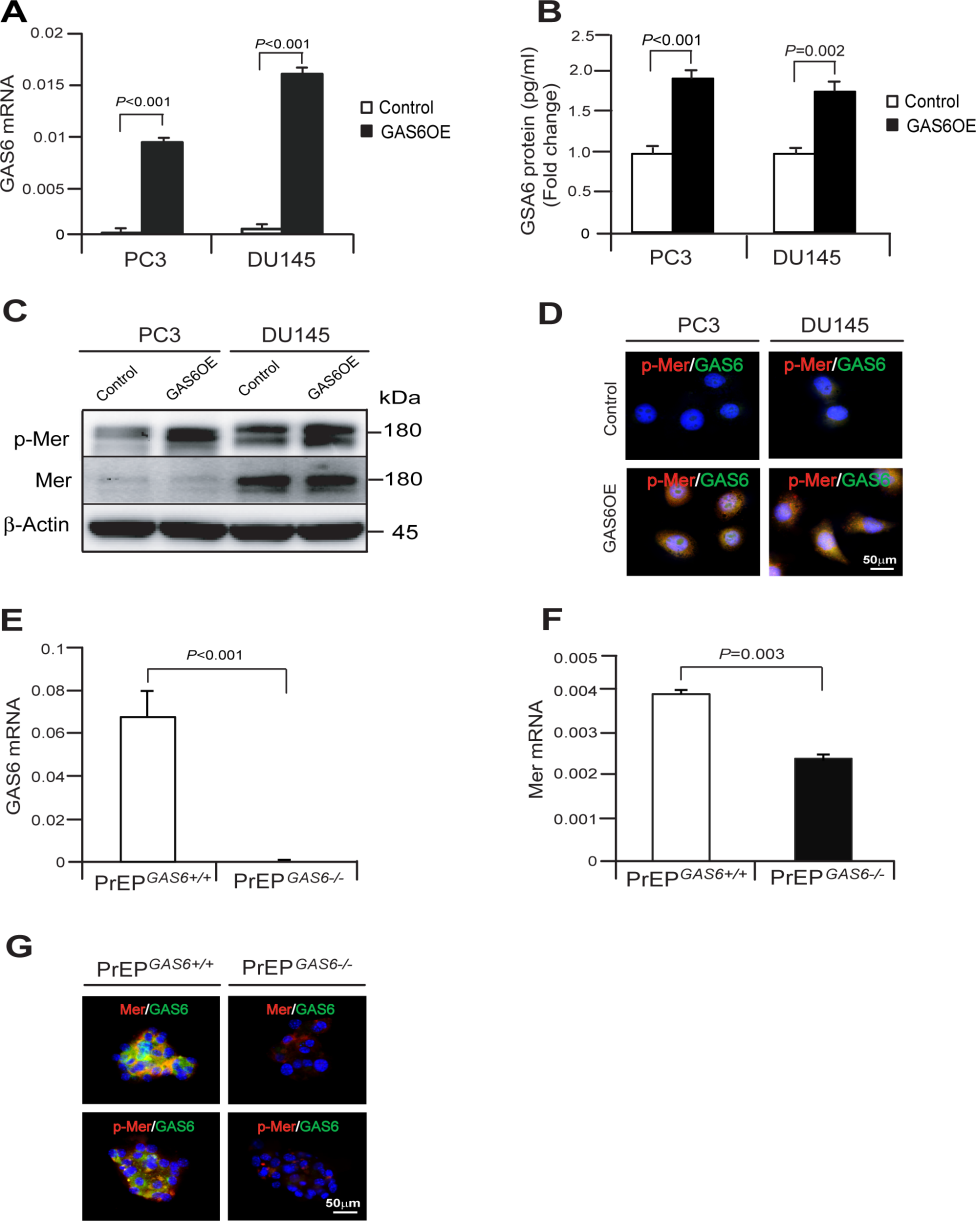
GAS6 overexpression activates the phosphorylation of Mer signaling in prostate epithelial cells or cancer cells **(A)** GAS6 mRNA expression as quantified by by real-time PCR in PCa^Control^ cells or PCa^GAS6OE^ cells. (**B**) GAS6 protein as quantified by ELISA in PCa^Control^ cells or PCa^GAS6OE^ cells. Data in Figure 4A, B are representative of mean with s.d. (Student's *t*-test). (**C, D**). PCa^Control^ cells or PCa^GAS6OE^ cells were starved for 24 hours and then exposed to 10% FBS of culture condition for 1 hour and phosphorylation of Mer was evaluated by (C) Western blot or (D) immunofluorescence staining. Bar = 50 μm. (**E, F**) GAS6 and Mer mRNA expression in the PrEP^*GAS6+/+*^ cells or PrEP^*GAS6−/−*^ cells by real-time PCR. **(G)** Mer (red)/GAS6 (green) or p-Mer (red)/GAS6 (green) in the PrEP^*GAS6+/+*^ cells or PrEP^*GAS6−/−*^ cells was evaluated by immunofluorescence staining. Bar = 50 μm.

To further explore whether GAS6 expression regulates a CSC phenotype in PCa cells, GAS6 was overexpressed in PC3 and DU145 cells (PC3^GAS6OE^ or DU145^GAS6OE^) (Figure 4A, 4B). When GAS6 was overexpressed in PCa cells, phosphorylation of Mer was highly activated in PCa^GAS6OE^ cells compared with PCa^Control^ cells as detected by Western blot and immunofluorescence staining (Figure 4C, 4D). Using GAS6 knockout mice, we also found that loss of Gas6 expression leads to a reduction of Mer expression and phosphorylation of Mer signaling. We found that Mer mRNA and protein expression were higher in the prostate epithelial cell spheres from PrEP^*GAS6+/+*^ than PrEP^*GAS6−/−*^ and Mer phosphorylation is correlated with levels of Mer expression (Figure 4E–4G). Collectively, these studies suggest that GAS6 expression regulates Mer receptor signaling in both normal prostate epithelial cells and cancer cells.

**Figure 5:**
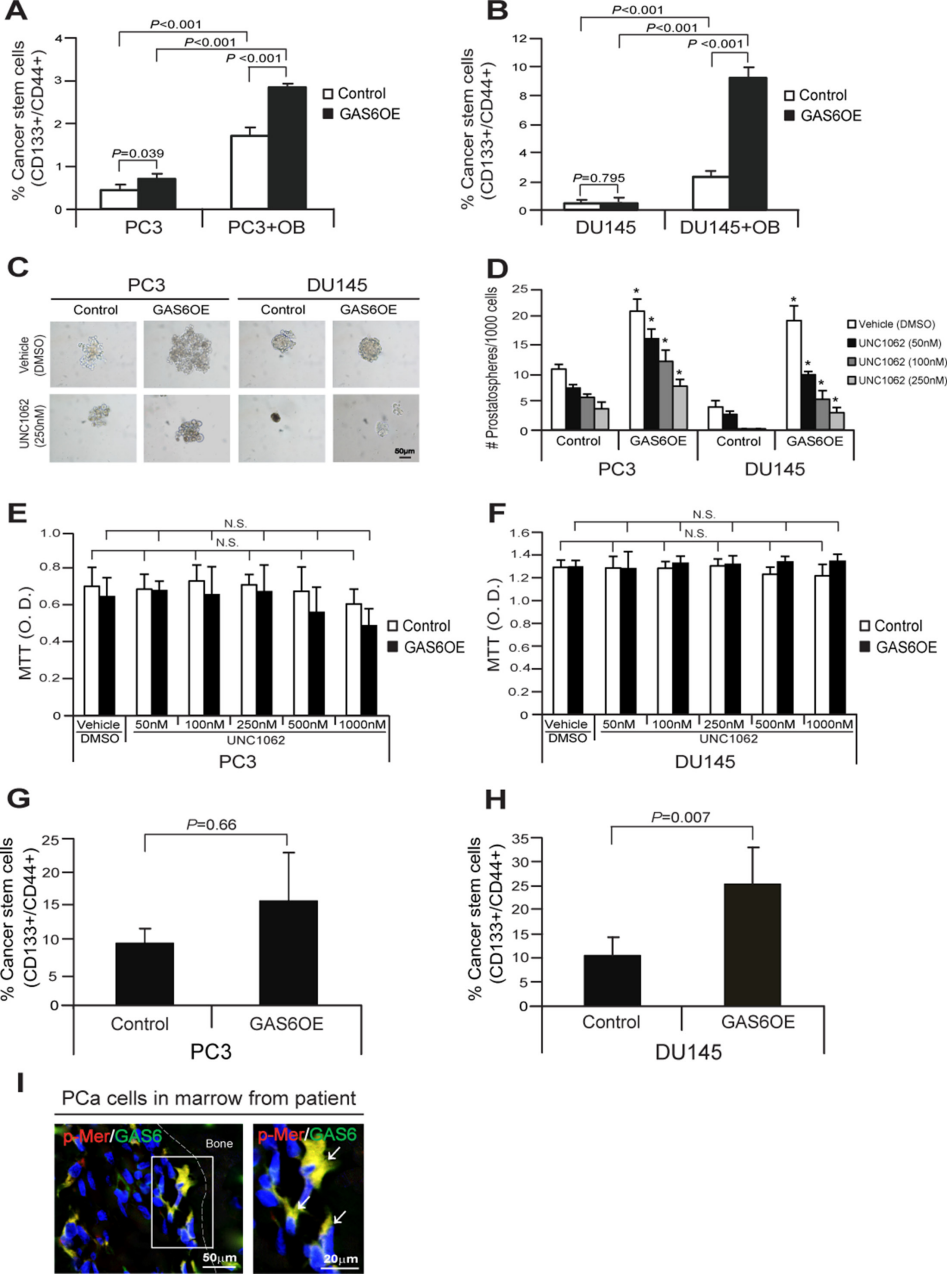
GAS6 overexpression increases CSCs through activation of Mer signaling in PCa cells (**A**, **B**) Percent of cells expressing the CSC phenotype in coculture of PCa^Control^ or PCa^GAS6OE^ with osteoblasts as quantified by FACS analysis. Data in Figure 5A, 5B are representative of mean with s.d. (Student's *t*-test). (**C**) The sphere formation of PCa^Control^ cells or PCa^GAS6OE^ cells in suspended sphere culture conditions following the treatment with a Mer inhibitor, UNC1062. Bar = 50 μm. (**D**) Quantification of prostatospheres in suspended sphere culture condition. *Denotes *p* < 0.05 between vehicle vs. a Mer inhibitor, UNC1062 treatments by Student's *t*-test. (**E**, **F**) MTT assays of PCa^Control^ cells or PCa^GAS6OE^ following the treatment with a Mer inhibitor, UNC1062 at 3 day cultures in adherent culture conditions. N.S. denotes no significance (*p* > 0.05) between vehicle vs. a Mer inhibitor, UNC1062 treatments by Student's *t*-test. (**G**, **H**) % CSC from DTC population in bone marrow at 24 hours after intracardiac injection of PCa^Control^ cells or PCa^GAS6OE^ in SCID mice (*n* = 5) as quantified by FACS analysis. Data are representative of mean with s.d. (Student's *t*-test). (**I**) Left panel: Immunofluorescence staining of p-Mer (red)/GAS6 (green) in PCa cells in bone marrow of a PCa patient. Blue, DAPI nuclearstain. Bar = 50 μm. Right panel: p-Mer (red)/GAS6 (green) positive cells (white arrows) in the magnification of the white rectangle from left panel. Bar = 20 μm.

We further examined whether CSC populations are correlated with GAS6 expression in PCa cells. PCa cells were segregated based upon expression of CD133 and CD44 from the cocultures with osteoblasts *in vitro*, and then quantified CD133+/CD44+ populations (Figure 2A). We found that CD133+/CD44+ populations were elevated in PCa^GAS6OE^ cells compared with PCa^Control^ cells from the cocultures with osteoblasts. However, CD133+/CD44+ populations were not altered in PCa^GAS6OE^ cells compared with PCa^Control^ cells in normal culture conditions (Figure 5A, 5B). Moreover, we examined whether the phosphorylation of Mer receptor results in alterations of sphere formation by PCa cells. We found that the number of prostatospheres that formed in culture were significantly increased in PCa^GAS6OE^ cells compared to PCa^Control^ cells, and critically, prostatosphere numbers were reduced following treatment with a Mer inhibitor, UNC1062 (Figure 5C, 5D). Likewise, we also observed smaller sphere sizes or disruption of sphere formation altogether from PCa cells following the treatment with UNC1062 in suspended sphere culture conditions (Figure 5C). Yet UNC1062 alone did not affect PCa cell viability under adherent culture conditions (Figure 5E, 5F). Based on these results, we examined how the bone marrow microenvironment responds to GAS6 overexpression by PCa cells and whether the CSC phenotype is affected *in vivo.* For these studies, the CD133+/CD44+ populations present in DTC populations were examined following isolation by FACS from mouse bone marrow 24 hours after *i.c.* injection of PCa^Control^ cells or PCa^GAS6OE^ cells (Figure 2D). CD133+/CD44+ populations were enhanced in the DTCs recovered from mouse bone marrow injected by PCa^GAS6OE^ cells compared with PCa^Control^ cells (Figure 5G, 5H). Finally, p-Mer expression was also detected in GAS6 expressing PCa cells within the bone marrow from a PCa patient as determined by immunofluorescence staining (Figure 5I). Collectively these data suggest that GAS6 overexpression acts to increase PCa CSCs via the activation of Mer receptor signaling in bone marrow.

## DISCUSSION

Recent studies suggest that GAS6 and Mer receptor signaling regulate the cell cycle status of cells as well as the induction of a stem cell phenotype in normal and cancer cells [14–19]. In this present study, we show that PCa CSCs express high levels of GAS6 within the bone marrow microenvironment. We also found that endogenous GAS6 expression is associated with Mer receptor expression in growth arrested G_1_ phase during the cell cycle, which is highly correlated with an increase of CSC populations of PCa cells. Moreover, we found that GAS6 overexpression activates the phosphorylation of Mer receptor, which increases CSC numbers in bone marrow microenvironment. Our results suggest that endogenous GAS6 increases PCa CSCs via the activation of Mer receptor signaling in bone marrow microenvironment, which may have important implications for targeting bone metastatic disease.

The homing and lodging of DTCs in the bone marrow and the maintenance of these cells in this microenvironment are essential steps to establish bone metastases [24]. Recent studies suggest that many hematopoietic and mesenchymal lineage cells in the bone marrow participate in the cellular and molecular events for the tumor progression and metastasis [24–35]. We recently reported that DTCs target and engage the hematopoietic stem cell (HSC) niche to establish metastases in marrow [33]. We have demonstrated that activation of Axl on PCa cells by GAS6 from osteoblasts in the bone marrow environment plays a critical role in establishing prostate tumor cell dormancy [6]. GAS6 signaling inhibits PCa proliferation, suggesting once DTCs enter the niche, interactions between GAS6 and its receptors may regulate PCa dormancy [6]. Interestingly, GAS6 expression is heterogeneous among bone locations; we further noted that the levels of GAS6 expression were significantly higher in the arm vs. leg bones [34]. These findings suggest that the environment of the arm bones is either not permissive or actively suppresses the growth of PCa compare to the leg bones [34]. Subsequently, we have demonstrated that a balance between the expression of Axl and Tyro3 is associated with a molecular switch between a dormant and a proliferative phenotype in PCa bone metastases [7]. In this investigation, we further explored the molecular mechanisms that would explain how DTCs become CSCs in bone marrow microenvironment. We found that the activation of Mer receptor signaling by endogenous GAS6 regulates the establishment and/or the maintenance of PCa CSCs within the bone marrow microenvironment. Observations that the enhanced expression of GAS6 mRNA in mammospheres from primary human mammary epithelial cell cultures [14] and reductions of neural stem-like cells in adult mammalian brain in GAS6 knockout mice [15] strongly supports our data that high level of endogenous GAS6 in DTCs regulates maintenance of CSCs. However, the exact mechanisms underlying how endogenous GAS6 expression occurs in DTCs within the bone marrow microenvironment requires further investigation.

Understanding the mechanisms of cell cycle regulation underlying CSC fate, such as self-renewal or proliferation, is one of the key pieces of data which is essential for our ability to better target CSCs in cancer therapy [36, 37]. Many cancer stem cells are either cycle very slowly or hardly at all and remain in G_0_; thus resistant to cell cycling-specific chemotherapy agents [38]. Abnormal regulation of cyclin-dependent kinases (Cdk) controls cell cycle progression, which may regulate self-renewal or proliferation of CSCs [39]. For example, recent studies show that knockdown of aldehyde dehydrogenase 1 family, member A1 (ALDH1A1) in ovarian cell lines results in dramatic decrease of KLF4 and p21 protein levels, thereby leading to S and G_2_ phase accumulation in ovarian cancer stem-like cells [40]. The G_0_ arresting cells in hepatocellular carcinoma Huh7 cells showed spheroid formation and 3-dimensional growth resulting in the marked tumorigenesis in NOD/SCID transplantation [41]. An additional study showed that CD44 knockdown causes breast cancer stem cells (BCSCs) to differentiate into non-BCSCs with lower tumorigenic potential, and CD44 knockdown BCSCs extended S phase similar to non-BCSCs [42]. The loss of PTEN can also lead to cell physiological changes, which sufficiently increases the self-renewing neural stem cells modulating G_0_– G_1_ cell cycle entry in glioblastoma [43]. Moreover, overexpression of an alternatively spliced isoform of the inhibitor of differentiation-1 (Id-1B) in PC3 PCa cells maintains cell quiescence, self-renewal, and cancer stem cell-like properties with increasing the proportion of cells in the G_0_–G_1_ phase of the cell cycle with high p27 levels and reduced p-Erk and cyclin A levels [44, 45]. In this regard, our results show that endogenous GAS6 expression and Mer receptor expression is associated with growth arrest in G_1_ by PCa cells, which correlates with an increase of CSC populations, suggesting a link between quiescent state and CSC population.

In this investigation, we explored the role that endogenous GAS6 and Mer receptor signaling play in establishing PCa CSCs within DTC population in bone marrow microenvironment. Previous works have demonstrated that Mer receptor signaling is involved in CSC activities under stem cell culture conditions in different cancers [18, 19]. Mer receptor expression is maintained in primary glioblastoma-derived tumor spheres and correlated with the expression of Nestin and Sox2 in a glioblastoma spheroid culture model, suggesting that Mer is crucial for the maintaining an undifferentiated state of cells [18]. Mer receptor inhibition by shRNA results in a reduction of colony formation and a decrease of tumor volume in human melanoma xenografts [19]. In fact, the melanoma cells treated with a Mer-selective tyrosine kinase inhibitor (UNC1062) lead to a reduction of Mer signaling, which stimulated apoptosis in culture, reduced colony formation in soft agar, and inhibited invasion [19]. Interestingly, both investigations pointed out that the autophosphorylation of Mer receptor signaling is likely to be crucial for maintenance of the rounded morphology of the spheres and cell invasive capacity in glioma cells [18] and melanoma cells [19]. In this investigation, we demonstrated that the phosphorylation of Mer was highly activated in PCa^GAS6OE^ cells vs. PCa^Control^ cells as well as PrEP^*GAS+/+*^ spheres vs. PrEP^*GAS6−/−*^ spheres. We also observed smaller sphere sizes and disruption of sphere formation in PCa^Control^ cells or PCa^GAS6OE^ cells following treatment with a Mer inhibitor. Together, the findings suggest that endogenous GAS6 expression is crucial to the CSC phenotype through the Mer receptor signaling.

In summary, this work provides evidence, which supports a crucial role for endogenous GAS6 and Mer receptor signaling in the establishment and/or maintenance of a CSC phenotype within the bone marrow microenvironment. Importantly, this work contributes to the understanding of the signaling mediators that facilitate tumorigenesis in PCa cells, and may have important implications for targeting metastatic disease.

## MATERIALS AND METHODS

### Cell culture

Human PCa cell lines (PC3, DU145, LNCaP) were obtained from the American Type Culture Collection (Rockville, MD). The metastatic subclone of LNCaP, C4–2B, was originally isolated from a lymph node of a prostate cancer patient with disseminated bony and lymph node involvement. GFP expressing PCa cell lines (PC3^*GFP*^ and DU145^*GFP*^ cells) were established by lenti viral transduction. Murine osteoblast cells were established as previously reported [20]. All prostate cancer cell lines were routinely grown in RPMI 1640 (Life Technologies, Carlsbad, CA), and murine osteoblast cells were grown in α-MEM (Life Technologies) supplemented with 10% fetal bovine serum (FBS, GEMINI Bio-Products, Sacramento, CA), 1% penicillin-streptomycin (P/S, Life Technologies) and maintained at 37°C, 5% CO_2_, and 100% humidity. Primary human prostate epithelial cells (HPEP; ATCC, PCS-440-010) was cultured in prostate epithelial cell basal medium (ATCC, PCS-440-030) with supplements (ATCC, PCS-440-040). The human prostate epithelial cell line RWPE-1 (ATCC, CRL-11609) was cultured in Keratinocyte-SFM (Life Technologies) with supplements (17005–042, Life Technologies).

### *In vitro* coculture

PCa cells were cultured on murine osteoblasts (OB) for 48 hours. Subsequently, the CD133+/CD44+ fraction was analyzed with a FACS Aria II High-Speed Cell Sorter by gating on HLA-ABC positive cells.

### Fucci-PC3 cells

To develop a method to monitor the cell cycle in prostate cancer cells, we transduced a human prostate cancer cell line, PC3 with lentiviruses containing the fluorescent ubiquitination-based cell cycle (Fucci) indicator vectors (Clontech, Mountain View, CA). Cells arrested in the G_1_ phase are fluoresced red due to the stability of the chromatin licensing and DNA replication factor 1 (CDT1)-Cherry reporter. Cells in G_2_ phase are green due to the stability of the of Geminin-Cyan reporter. S phase cells are double-positive for the reporters, fluorescing yellow. M phase is colorless due to the destruction of both Geminin-Cyan and CDT1-Cherry [21]. pRetroX-G_1_-Red vector (cat. 631463, Clontech) and pRetroX-SG_2_M-Cyan vector (cat. 631462, Clontech) were packaged into lentivirus at the University of Michigan Vector Core Facility. Lentiviral pRetroX-G_1_-Red vector and lentiviral pRetroX-SG_2_M-Cyan vector were coinfected into PC3 cells. Infected cells were selected for 7 days in media containing 1 μg/ml Puromycin and analyzed by FACS. Fucci-PC3 cells were further verified by FACS analysis or Western blots.

### GAS6 overexpression

Human GAS6 overexpression plasmid vector (Ex-Z6826-Lv105) and control vector, (Ex-NEG-Lv105) (GeneCopia, Rockville, MD) were packaged with lentivirus at the University of Michigan Vector Core Facility. Lentiviral human GAS6 vector or control vector were infected into PCa cells (PC3 and DU145). Infected cells were selected for 7 days in media containing 1 μg/ml Puromycin and analyzed by real-time PCR or Elisa. Human GAS6 overexpression was verified by real-time PCR and Elisa.

### ELISA

Control PCa cells (PC3^Control^ or DU145^Control^) or GAS6 overexpressed PCa cells (PC3^GAS6OE^ or DU145^GAS6OE^) (1 × 10^5^) were seeded onto 12-well culture plates, and then cultured for 48 hours. The conditioned media were collected. An antibody sandwich ELISA was used to evaluate GAS6 expression in the conditioned media by the following to the directions of manufacturer (cat. DY885, R & D Systems, Minneapolis, MN). GAS6 level were normalized to total cell numbers.

### Cancer stem cell (CD133+/CD44+ phenotype) analysis

Control PCa cells (PC3^Control^ or DU145^Control^) or GAS6 overexpressed PCa cells (PC3^GAS6OE^ or DU145^GAS6OE^) (1 × 10^5^) were seeded onto 12-well culture plates, and then cultured for 48 hours. PCa cells were incubated with PE-anti-CD133 antibody (cat. 130-080-901, Miltenyi Biotec, San Diego, CA) and APC-anti-CD44 antibody (cat. 559942, BD Biosciences, San Jose, CA) for 20 min at 4°C. The CD133+/CD44+ fraction was analyzed with a FACS Aria High-Speed Cell Sorter (BD Biosciences).

### Quantitative RT-PCR

Total RNA was extracted from cells using the RNeasy mini or micro kit (Qiagen, Valencia, CA) and converted into cDNA using a First-Strand Synthesis Kit (Invitrogen). Quantitative PCR was performed on an ABI 7700 sequence detector (Applied Biosystems) using TaqMan Universal PCR Master Mix Kit (Applied Biosystems) according to the directions of manufacturer. TaqMan MGB probes (Applied Biosystems) were as follows: GAS6 (Hs00181323_m1), Mer (Hs00179024_ m1). β-actin (Hs01060665_g1) was used as an internal control for the normalization of target gene expression.

### Immunostaining

Cells, tumor sections and long bone sections were used for immunostaining. Cells were fixed and permeabilized with Perm/Wash Buffer (cat. 554723, BD Biosciences). Tumor sections were blocked with Image-iT FX signal enhancer for 30 min and incubated for 2 hours at room temperature with primary antibodies combined with reagents of Zenon Alexa Fluor 488 (green) or 555 (red) labeling kit (Invitrogen). Human GAS6 (cat. AF885, R & D System), Mer (cat. ab110108, Abcam, Burlingame, CA), p-Mer (cat. NB300-690, Novus Biologicals, Littleton, CO), CD133 (cat. 130–090–423, Miltenyi Biotec), CD44 (cat. ab6124, Abcam), CK18 (cat. ab7797, Abcam) antibodies were used as primary antibody. After washing with PBS, the slides were mounted with ProLong Gold antifade reagent with DAPI (Invitrogen). Images were taken with Olympus FV-500 confocal microscope. Human prostate tissue microarrays (TMAs) were purchased from US Biomax, Inc. (Rockville, MD). Tumors were graded using stage progressing system. Anti-human GAS6 and CK18 antibodies were applied for coimmunostaining. Staining intensity was ranked on a scale from 1 to 4 (1, negative; 2, weak; 3, moderate; and 4, strong intensity staining).

### Western blot

PCa cells were cultured in RPMI 1640 with 10% FBS and 1% P/S. Whole cell lysates were prepared from cells, separated on 4–20% Tris-Glycine gels and transferred to PVDF membranes. The membranes were incubated with 5% milk for 1 hour and incubated with primary antibodies overnight at 4°C. Primary antibodies used were as follows: monoclonal anti-Cyclin A2 (1:1,000 dilution, cat. 4656, Cell Signaling, Danvers, MA), monoclonal anti-CDK2 (1:1,000 dilution, cat. sc-63, Santa Cruz, Santa Cruz, CA), monoclonal anti-p21 (1:1,000 dilution, cat. 2947, Cell Signalng), monoclonal anti-Mer (1:1,000 dilution, cat. 4319, Cell Signaling), p-Mer (1:1000 dilution, cat. ab14921, Abcam). Blots were incubated with peroxidase-coupled anti-rabbit IgG secondary antibody (cat. 7074, 1:2,000 dilution, Cell Signaling) for 1 hour, and protein expression was detected with SuperSignal West Dura Chemiluminescent Substrate (cat. Prod 34075, Thermo Scientific, Rockford, IL). Membranes were reprobed with monoclonal anti-α-Tubulin (1:1,000 dilution, cat. 2125, Cell Signaling) or monoclonal anti-β-actin antibody (1:1,000 dilution; cat. 4970, Cell Signaling) to control for equal loading.

### Prostatosphere culture and assay

The control PCa cells (PCa^Control^) or GAS6 overexpressed PCa cells (PCa^GAS6OE^) were dissociated to single cells by standard trypsinization and washed three times with PBS. The cells were plated in stem cell culture medium (DMEM: F12 plus 10 ng/ml bFGF, 20 ng/ml EGF, 5 mg/ml insulin, and 0.4% BSA) supplemented with 1% KO serum replacement (cat. 10828–028, Invitrogen) [22] at a density of 1,000 cells/ml in low attachment 6 well culture plates. A Mer inhibitor, UNC1062 (cat. AOB4488, Aobious, Gloucester, MA) was treated to the cells. Seven day old spheres were enumerated as cell clusters comprised of >30 cells. Mouse prostatosphere formation assay was performed following the protocol previously published by Lukacs et al. [23]. Prostates were harvested from aged matched wild-type (PrEP^*GAS6+/+*^) or GAS6 knockout (PrEP^*GAS6−/−*^) mice, then minced and digested with Collagenase A (cat. 10103586001, Roche, Nutley, NJ) for 2 hours on a shaker at 37°C. Cells were spun down at 400 g for 5 min. Supernatant was removed and cells were incubated in 2 ml of trypsin at 37°C for 5 min. Cells were then pelleted and suspended in media containing 500 U of DNase I to inactivate the trypsin. The pellet was then passed through a 20 G syringe. Cells were filtered through a 40 μm filter. Cells (PrEP^*GAS6+/+*^ or PrEP^*GAS6−/−*^) (2.5 × 10^5^) were plated in prostate epithelial sphere culture media in low-attachment 6-well plates. Seven to ten day old spheres were used for the evaluation of gene or protein expression.

### *In vivo* analysis of disseminated prostate cancer stem cells

All experimental animal procedures were performed in compliance with the institutional ethical requirements and approved by the University of Michigan Committee for the Use and Care of Animals (UCUCA). PCa^Control^ or PCa^GAS6OE^ cells (1 × 10^6^ cells) were injected into male CB.17. SCID mice (4–6 weeks of age, Charles River, Wilmington, MA) by intracardiac (*i.c.*) injection. Bone marrow cells were flushed from the femorae and tibias 24 hours later and murine hematopoietic lineages were depleted with Miltenyi lineage depletion kit (cat.) using AutoMACS (Miltenyi Boitecs). The enriched cells were incubated with a FITC-anti-HLA-ABC, PE-anti-CD133, and APC-anti-CD44 antibodies. Thereafter, the CD133+/CD44+ and CD133–/CD44– fractions of the HLA positive cells were analyzed with a FACS Aria II High-Speed Cell Sorter (Becton Dickinson, Franklin Lakes, NJ). In some cases, the CD133+/CD44+ and CD133–/CD44– fractions were sorted for gene expression of GAS6 and Mer.

### Statistical analyses

Results are presented as mean ± standard deviation (s.d.). Significance of the difference between two measurements was determined by unpaired Student's *t*-test, and multiple comparisons were evaluated by the Newman-Keuls multiple comparison test. Values of *p* < 0.05 were considered significant.
